# Clinical characteristics and outcome of patients with status myoclonus following out of hospital cardiac arrest

**DOI:** 10.1186/2197-425X-3-S1-A198

**Published:** 2015-10-01

**Authors:** MI Ruknuddeen, V Rajajee, R Ramadoss, L Grzeskowiak, R Rajagopalan

**Affiliations:** Lyell McEwin Hospital, Intensive Care Medicine, Adelaide, Australia; University of Michigan, Neurosurgery and Neurology, Michigan, United States; University of Adelaide/Lyell McEwin Hospital, Intensive Care Medicine, Adelaide, Australia; University of Adelaide/The Robinson Research Institute, Adelaide, Australia; Sundaram Medical Foundation, Intensive Care Medicine, Chennai, India

## Introduction

Generalized status myoclonus following out of hospital cardiac arrest (OHCA) has been associated with poor prognosis^1^. Many patients die and majority of those who survive remain in a vegetative state^2^. There is paucity of data on the clinical characteristics and outcome of patients with status myoclonus treated with hypothermia from resource-limited centres in South Asia.

## Objectives

To study the clinical characteristics and outcome of patients with status myoclonus following out of hospital cardiac arrest and treated with hypothermia.

## Methods

Retrospective chart review of patients surviving > 24 hours following OHCA in an urban community hospital in South India, between January 2006 and December 2012. All patients received therapeutic hypothermia (32-34°C) for 24 hours. Data on the clinical characteristics of patients developing status myoclonus (clinical myoclonic status epilepticus) and their outcome (survival, morbidity and mortality) were studied in detail. Based on the cerebral performance category (CPC) score at hospital discharge, the neurological outcome of patients was classified into favorable (CPC1, 2) and unfavorable (CPC 3,4).

## Results

Out of the 124 patients surviving > 24 hours, 52 (42%) developed status myoclonus. Baseline and general characteristics were similar in patients with and without status myoclonus (Table 1). Majority of patients with status myoclonus had absent brainstem reflexes [(pupillary, corneal and cough) 92% vs. 62%, P < 0.001] and motor response worse than flexion (100% vs. 71%, P < 0.001) on day 3 post-cardiac arrest (Table 2). None of the patients with status myoclonus had favorable neurological outcome (50 patients died and 2 patients were discharged in vegetative state, CPC 4). Out of the 72 patients without status myoclonus, 18 (25%) patients had favorable neurological outcome (CPC1). Out of the 104 patients who died, 53 (51%) were following withdrawal of life sustaining treatment (WLST) and majority (37/53) of these patients had status myoclonus (74% vs. 30%, P < 0.001).

## Conclusions

Status myoclonus following out of hospital cardiac arrest was associated with unfavorable outcome. Majority of deaths in patients who developed status myoclonus was secondary to WLST, which may indicate self-fulfilling prophecy.

**Table 1:** General Characteristics.

Characteristics	Status Myoclonus Present N= 52	Status Myoclonus Absent N= 72	P Value
Duration of cardiac arrest >20 minutes N (%)	50 (96.2)	60 (87.0)	0.113
Charlson comorbidity index Median (IQR)	3 (2-6)	5 (2-6)	0.328
APACHE II score Median (IQR)	34 (30-37.5)	35 (31-40)	0.420
Sex N (%) Male Female	33 (63.5) 19 (36.5)	44 (63.8) 25 (36.2)	0.972
Age Median Years (IQR)	60 (47.5-67.5)	63 (54-70)	0.250
Metabolic Acidemia at presentation N (%) PH <7.0	49 (94.2)	63 (91.3)	0.544
Etiology of cardiac arrest: Non-cardiac N (%)	36 (69.2)	37 (53.6)	0.082
Non-shockable rhythm N (%)	50 (96.2)	59 (85.5)	0.052

**Table 2:** Neurology, Morbidity and Mortality.

Neurological Characteristics	Status Myoclonus Present N= 52	Status Myoclonus Absent N= 72	P value
Absent brainstem reflexes on day 3, post-cardiac arrest N (%)	47 (90.4)	43 (62.3%)	< 0.001
Motor response worse than flexion, day 3 post-cardiac arrest N (%)	52 (100)	49 (71)	< 0.001
CPC 1 N (%)	0	18 (25%)	< 0.001
CPC 4 (N %)	2 (4)		
Mortality N (%) 104 (84)	50 (96)	54 (75)	< 0.001
Death Due to underlying illness N (%) 51 (49)	13 (26)	38 (70.4)	< 0.001
Death following withdrawal of life sustaining treatment N (%) 53 (51)	37 (74)	16 (29.6)	< 0.001
